# Rapid Classification of Hairtail Fish and Pork Freshness Using an Electronic Nose Based on the PCA Method

**DOI:** 10.3390/s120100260

**Published:** 2011-12-28

**Authors:** Xiu-Ying Tian, Qiang Cai, Yong-Ming Zhang

**Affiliations:** 1 Instrument Lab, Yangtze Delta Region Institute of Tsinghua University, Jiaxing 314006, Zhejiang, China; E-Mail: xiuying.tian@hotmail.com; 2 Department of Life and Environmental Science, Shanghai Normal University, Shanghai 200237, China; E-Mail: zhym@shnu.edu.cn

**Keywords:** electronic nose, sensor, hairtail fish, pork, principal component analysis (PCA), total volatile basic nitrogen (TVBN), freshness, shelf life

## Abstract

We report a method for building a simple and reproducible electronic nose based on commercially available metal oxide sensors (MOS) to monitor the freshness of hairtail fish and pork stored at 15, 10, and 5 °C. After assembly in the laboratory, the proposed product was tested by a manufacturer. Sample delivery was based on the dynamic headspace method, and two features were extracted from the transient response of each sensor using an unsupervised principal component analysis (PCA) method. The compensation method and pattern recognition based on PCA are discussed in the current paper. PCA compensation can be used for all storage temperatures, however, pattern recognition differs according to storage conditions. Total volatile basic nitrogen (TVBN) and aerobic bacterial counts of the samples were measured simultaneously with the standard indicators of hairtail fish and pork freshness. The PCA models based on TVBN and aerobic bacterial counts were used to classify hairtail fish samples as “fresh” (TVBN ≤ 25 g and microbial counts ≤ 10^6^ cfu/g) or “spoiled” (TVBN ≥ 25 g and microbial counts ≥ 10^6^ cfu/g) and pork samples also as “fresh” (TVBN ≤ 15 g and microbial counts ≤ 10^6^ cfu/g) or “spoiled” (TVBN ≥ 15 g and microbial counts ≥ 10^6^ cfu/g). Good correlation coefficients between the responses of the electronic nose and the TVBN and aerobic bacterial counts of the samples were obtained. For hairtail fish, correlation coefficients were 0.97 and 0.91, and for pork, correlation coefficients were 0.81 and 0.88, respectively. Through laboratory simulation and field application, we were able to determine that the electronic nose could help ensure the shelf life of hairtail fish and pork, especially when an instrument is needed to take measurements rapidly. The results also showed that the electronic nose could analyze the process and level of spoilage for hairtail fish and pork.

## 1. Introduction

Food safety is a fundamental and legal requirement. Fish and pork are very popular in many countries because of their good flavor and great health benefits [1]. Among meat and protein sources for human consumption, fish is the most perishable and pork is also easily spoiled, so freshness control for fish and pork has received a great deal of attention from the food industry in recent years [2]. Shelf life is defined as the period in which a food product remains safe and fit for consumption under defined storage conditions [3]. Scientists have been constantly searching for improved methods to preserve or extend the shelf life of fish and pork. The quality of fish degrades due to both microbial spoilage and biochemical reactions that occur during handling and storage. Fish and pork offered for sale must be safe, although they do not necessarily have to be of the highest quality. Thus, a quick assessment method for fish and pork muscle quality during storage is necessary.

Consumption of spoiled hairtail fish (*Trichiurus lepturus*) or pork products could result in serious health hazards. Odor is one of the most important indicators of fish or pork freshness and develops with time during storage [4]. However, the traditionally methods of sensory panels or GC/MS are time-consuming and costly [5,6]. Thus, studies to develop an “electronic nose” for fish or pork freshness measurement and safety application have been conducted or are currently in progress [7–9].

The reference method currently used for determining the spoilage status of meat is the analysis of the total count of aerobic bacteria and total volatile basic nitrogen (TVBN). These methods are good indicators of product safety and can be employed in many cases to define the desired product quality. However, the two methods have several drawbacks, such as complex and time-consuming operation steps. For instance, the incubation period of the bacteriological method is 1 to 2 days [10], and the errors and biases in the analysis results of various TVBN procedures are inevitably increased [11]. Despite these drawbacks, the results obtained from bacteriological and TVBN analyses can be used for developing alternative methods, such as an electronic nose system.

An “electronic nose” is a system originally created to mimic the function of human nose. There are three primary components in an electronic nose: an array of chemical gas sensors with broad and partly overlapping selectivity that measure volatile compounds, a signal-preparation system, and a pattern-recognition system [12,13]. An electronic nose is characterized by high sensitivity, reproducibility, and reliability. It is also highly efficient, with a short reaction and recovery time as well as a low cost. An electronic nose can be used to measure and monitor odor anywhere, suggesting its many different applications, for example, in the food and beverage industry. This device can be regarded as an interesting tool for quick quality tests in various food applications [14].

PCA, a basic classification technique, is a powerful, linear, and unsupervised pattern-recognition method that is often successfully used in gas sensor applications [15,16] and for investigating the performance of the electronic nose system in the spoilage classification of fish and meat. A large number of studies have applied the electronic nose to monitor changes in volatile compounds during the storage of fish [17] and to assess the freshness of fish [18]. Portable electronic noses have even been developed based on different sensors [19–21]. Yano *et al.* [22] proved that a microbial sensor was useful for the quality control of pork freshness.

Considerable work on separately assessing fish and pork freshness has already been conducted. However, most of these studies were performed in a laboratory and were based on a single material. Therefore, further research is necessary. The potential exists to develop an electronic nose for multitudes of products that would be both functional and convenient. Based on laboratory research, using the electronic nose to solve practical issues is the key. The purpose of the current research is to evaluate the performance of an electronic nose as an effective instrument that could rapidly classify hairtail fish and pork freshness and to apply this system to measure freshness of the products. In the current study, we first built an electronic nose based on the MOS gas sensors for the specific gases assumed to be fish and pork degradation products, such as trimethylamine (TMA), dimethylamine (DMA), and ammonia [17,23]. We then tested the freshness of hairtail fish and pork stored at 15, 10, and 5 °C using this electronic nose. The measurements using the electronic nose were compared with classical TVBN measurements. Subsequently, we applied the electronic nose in actual supermarket shelves based on the patterns developed in the laboratory.

## 2. Experimental

### 2.1. Experiments in the Laboratory

#### 2.1.1. Electronic Nose Set-Up

**Figure 1.**
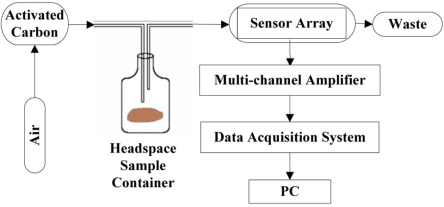
Schematic diagram of the electronic nose system.

Spoilage odor from microbial growth and oxidation, which results in the degradation of hairtail fish or pork, was sensed using a simple and cheap electronic nose prototype developed in our laboratory. The electronic nose prototype is shown in Figure 1.

The sensor array, multi-channel amplifier, and data acquisition system developed in-house are placed in a box. Electronics, an A/D converter, and a microprocessor that reads the measurements and sends these data to the PC are also placed in the box. A miniature sampling pump is positioned in the case to ensure gas circulation. The measurement program is run on a PC. The sensor array reacts to the signal from each sensor resistance when a given sample is present. We use the multi-channel amplifier to magnify the signal to record it conveniently and accurately. The data-acquisition system interacts continuously with the environment. The embedded software controls the circuits and reads the sensor data synchronously. Computer software drivers have been especially designed to meet the needs of the data acquisition system of the electronic nose. These drivers are also used to collect and identify the sensor array responses for further data processing. Air filtered by the activated carbon is used to clean the headspace sample container and polytetrafluoroethene (PTFE) chamber when the sample is absent to prepare for the next measurement. PTFE is a thermoplastic polymer, which is a white solid at room temperature, with a density of about 2.2 g/cm^3^. Its melting point is 327 °C (621 °F) and coefficient of friction is 0.05 to 0.10, which is the third-lowest of any known solid material. Because of PTFE’s chemical inertness and no “memory”, it can be used as a seal. PTFE’s resistance to van der Waals forces means that little substance can stick on its surface. So FTFE is chosen as the material for chamber.

**Figure 2.**
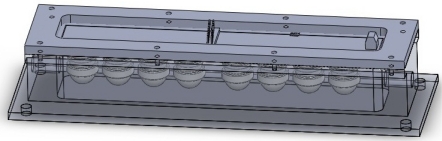
Schematic diagram of the PTFE chamber.

As shown in Figure 2, the sensor array is located in the PTFE chamber, which includes eight tin oxide based MOS gas sensors (MQ type, Hangzhou Ke Na Sensors Inc., and TGS type, Figaro Inc.) and two commercial SHT series sensors (SENSIRION Inc.) for temperature and relative humidity control. Each sensor has a certain degree of affinity towards a specific chemical or volatile compound. The identification codes of the sensors used, where the target gases suggested by the manufacturer are indicated, are the following: MQ135 (S1, NH3, ethanol, and smoke), TGS 825 (S2, H_2_S), TGS 824 (S3, NH_3_, and amines), MQ3 (S4, ethanol vapor), TGS 826 (S5, NH_3_, and amines), TGS 880 (S6, water vapor in cooking process), TGS 800 (S7, gasoline exhausts), TGS 822 (S8, alcohols and organic solvent vapor), SHT10 (measurement range: 0% to 100% relative humidity, −40 °C to 123 °C), and SHT10 (measurement range: 0% to 100% relative humidity, −40 °C to 123 °C).

#### 2.1.2. Sample Preparation

Three batches of fresh hairtail fish and pork from a pig’s hind leg were obtained from a local supermarket. These products were immediately brought to the laboratory in portable refrigerated containers. Ice was added after receiving the samples. Hairtail fish were washed by immersion in ice-cold water. The heads and tails of the fish were removed and cut into pieces of the same weight (approximately 50 g). The pork, upon arriving at the laboratory, was washed several times through immersion in ice-cold water, and the skin and fat were removed. After drying, the pork was cut into pieces of the same weight (approximately 50 g). The two types of samples were immediately placed individually in clean plastic bags (bags for freezing food) that were subsequently placed in a refrigerator. The temperature of the refrigerator was respectively set to 15, 10, and 5 °C to measure different indexes of freshness. The fish samples stored at 15 °C were measured twice a day for 3 days. The samples stored at 10 °C were measured at one-day intervals for 4 days, and the samples stored at 5 °C were measured once a day for 5 days. The pork samples stored at 15 °C were measured twice daily for 3 days. The samples stored at 10 °C were analyzed daily for 5 days, and the samples stored at 5 °C were measured once a day for 7 days. To obtain a more accurate result, every measurement was repeated six times. Temperature and humidity were also recorded. For each measurement, two same samples were taken from the refrigerator to undergo TVBN and microbiological analysis, and three same samples were employed for electronic nose analysis. This procedure was repeated until the experiment was finished.

#### 2.1.3. Sensory Evaluation to Hairtail Fish

At present, fish and seafood freshness measurement largely relies on the sensory assessment of freshness attributes. According to a certain grading scheme, these qualities are compiled to produce a quality index. Sensory evaluation involves the use of sight to evaluate skin appearance, the color, and the global aspect of the eyes, tactile to test flesh firmness and elasticity, and olfaction to smell gill odor [24].

#### 2.1.4. TVBN Measurements

TVBN (mg of N/100 g of whole fish or pork) was measured according to the appropriate Chinese standards [25]. A sample of each product (50 g) was taken from the refrigerator and processed, which included the removal of fat, bone, and tendon. Subsequently, the sample was homogeneously cut into smaller pieces weighing approximately 10 g and placed inside a tapered bottle. A total of 100 mL water was added into the tapered bottle, which was vibrated constantly for 30 min. Finally, the compound of the bottle was filtered, and the filtrate was kept in the refrigerator. The entire process must be sterile. The tapered bottle contained boric acid absorbing liquid (10 mL, 20 g/L) and five or six drops of mixed indicator, formed by a mixture of sub-methylene blue (1 g/L) and methyl red-ethanol (2 g/L). An indicator was placed above the condenser. The condenser pipe must be immersed in the absorbing liquid. A total of 5 mL filtrate was placed into the reaction chamber of the distiller, to which MgO (5 mL, 10 g/L) was added. Distillation was performed for 5 min after venting with steam. The boric acid absorbing liquid was titrated by hydrochloric acid (0.01 mol/L) until it turned bluish violet. A blank reagent experiment was also performed. The TVBN formula was calculated as follows:

(1) $$X=\frac{(V_{1}-V_{2})\times c\times A}{m\times 5/100}\times 100$$

where X is the TVBN of the samples (mg/100 g), V_1_ is the consumption amount of hydrochloric acid by the titrated boric acid absorbing liquid (mL), V_2_ is the consumption amount of hydrochloric acid by the titrated blank absorbing liquid (mL), c is the concentration of the hydrochloric acid (mol/L), and A is the mass of the nitrogen amount with 1 mL hydrochloric acid standard titration solution (1 mol/L) (mg). In this equation, A = 14, and m is the mass of the sample (mg) being measured.

#### 2.1.5. Aerobic Bacterial Plate Counts

Aerobic bacterial plate counts were performed to show the number of aerobic bacteria found in or on the fish or pork muscle at various stages of degradation. The method used to perform the plate counts conform to a related Chinese procedure [26].

A sample of each product (50 g) was taken and cut aseptically into small pieces. A sample (25 g) was placed in a sterile glass bottle containing 225 mL sterilized physiological saline and was made into 1:10, 1:100, and 1:1,000 uniform dilutions. An aliquot (1 mL) of each diluted concentration was transferred to two replicated sterilized Petri dishes, and 15 mL nutrition agar medium at 46 °C was placed into the sterilized Petri dishes. The blank reagent experiment was also performed by adding the same nutrition agar medium into the sterilized Petri dishes containing 1 mL diluents. The plates were incubated at 36 °C ± 1 °C for 48 h ± 2 h after the nutrition agar solidification. The total aerobic bacterial plate counts were obtained by enumerating the colonies present. The results were expressed as the cfu/g of the sample.

#### 2.1.6. Measurements with the Electronic Nose

The response time of the sensors was approximately 60 s, but the system needed time to equilibrate. The measurements in a flow of pure air filtered by the activated carbon were continued for 30 min to observe the sensor signal and to allow time for equilibration. The measurement could start when the final values of all the sensors was equilibrated. Approximately 15 min was needed to allow the sensors to recover and reach the initial value after an exposure time of a sample, such as spoiled hairtail fish or pork. Thus, each measurement comprised two phases. In the first phase, which lasted 15 min, the response of the sensors in a flow of pure air filtered by the activated carbon was acquired. This phase is essential because it allowed the gas sensors to reach a stable and reproducible resistance, which was considered their baseline resistance (or their initial state). In the second phase, the response of the sensors was acquired in 1 min when a flow of volatile compounds is emitted from the dynamic headspace of the hairtail fish or pork samples. The dynamic headspace is fluxed into the electronic nose sensor chamber, using the sampling pump. At the end of phase two, a new measurement process could be initiated immediately by restarting phase one. Exhaust was absorbed by an aqueous solution.

Five pieces of fish or pork samples were taken from the refrigerator, placed in a sterilization Petri dish and heated in a thermostatic water bath at 55 °C for 5 min before being introduced into the sample container [27].

The measurement technique for the analysis of volatile compounds from hairtail fish or pork using the gas sensor instrument is based on a dynamic headspace system that analyzes the direct dynamic headspace of samples stored in the headspace sample container at room temperature. The flow of the sampling pump was set to 3 L/min.

#### 2.1.7. Feature Extraction and Analysis

Data acquisition was controlled using a laptop, and the digital signal of each sensor was recorded as a function of time. The feature used for data analysis is extracted from the temporal responses of the sensor array. We wanted to extract more accurate information from each experiment. Thus, we used features that could characterize the digital signal of each sensor. Two representative features were extracted from the response signal, namely:

- R_0_, which is the initial resistance of a sensor calculated as the average value of its resistance during the first 15 min of a measurement when in the absence of the sample, before running every experiment with the samples, referred to as the baselines in the current work.
- R_0-stable_: the steady-state resistance calculated as the average value of its resistance during the last 1 min of a measurement, when the piece of sample is introduced into the sample container and recording of the signals was started (time zero) until no time variation was observed for all the sensors with the time evolution. This is referred as the stable in the current work.
- S (S = S1, S2, S3, S4, S5, S6, S7, S8, H1, H2): the real resistance calculated as:
  (2) $$S_{\mathrm{ij}}=R_{0-\mathrm{stable}}-R_{0,\mathrm{ij}}$$
  where i (i = 1, ……, N) is the number of samples, j (j = 1, ……, N) is the identification codes of sensors, and S_ij_ is referred to as the response of a sensor in the current work.

Traditionally, the dataset was pre-processed using standard procedures, such as mean-centering, standardization, or matrix normalization, depending on the different pattern recognition methods employed. Pre-processing the resulting data matrix using an automated process via a written-in-house MATLAB 7.0 program, and two aforementioned features were extracted from the data of each measurement.

The primary purpose for using pattern-recognition methods in this particular application was to estimate the performance of the electronic nose at classifying the freshness of hairtail fish and pork samples, which had undergone cold storage at different temperatures, to identify their shelf life. Performance was assessed by employing a statistical method. The aim of PCA was to allow a visual approach to the problem in a reduced representative space defined by principal components. Thus, linear combinations were calculated with the original representative variables, and the information in these original variables was expressed in lower new variables called principal components. These principal components were selected to contain the maximum of the data variance and were orthogonal. The percentage of the data variance contained in each principal component was given by the corresponding eigenvalue. Finally, all the redundancies were removed, and the new scores were calculated for each principal component and measurement in the database [2]. Before PCA, Kaiser-Meyer-Olkin (KMO) and Bartlett’s test of sphericity was performed. PCA was fitted for a KMO value over 0.7 and for a load coefficient exceeding 0.5. Being unsupervised, PCA groups (separate) samples together according to similarities (differences) in input data (*i.e*., features extracted from the sensor response). Principal components of the sensor array were obtained by PCA, and the contribution of each factor to the principal components was also analyzed.

The expression of the principal components is as follows:

(3) $$\mathrm{PC}=\left[\begin{array}{c} {\mathrm{PC}}_{1}\\ \begin{array}{l} .\\ .\\ . \end{array}\\ {\mathrm{PC}}_{j} \end{array}\right]=A\cdot X=\left[\begin{array}{ccc} a_{1,1} & ... & a_{1,10}\\ ... & ... & ...\\ a_{K,1} & ... & a_{k,10} \end{array}\right]\cdot {\left[\begin{array}{c} X_{1}\\ ...\\ X_{10} \end{array}\right]}^{T}$$

Original vector X = [X_1_, X_2_, ……, X_10_]^T^ = [S_1_ S_2_ S_3_ S_4_ S_5_ S_6_ S_7_ S_8_ H_1_ H_2_]^T^, where j is the serial number of the principal component, i is the serial number of the sensor, K is the dimension of data, and a_ki_ is the coefficient.

### 2.2. Practical Application

#### 2.2.1. Site Definition and Instrument

Suguo is a large supermarket chain for the Blacksmith Camp community in Nanjing. We placed our electronic nose on the supermarket’s hairtail fish and pork shelves.

#### 2.2.2. Sample Preparation

In the supermarket, the test subjects were hairtail fish and pork from a pig’s hind leg available at the Suguo supermarket. The storage conditions of the test subjects were similar to those of other products on the supermarket shelves. The same day, hairtail fish (saved in the ice) and pork were preserved on the shelves. Hairtail fish was stored in special ice water, and pork was stored in the refrigerator after the supermarket closed at night. This procedure was repeated until the hairtail fish and pork spoiled.

#### 2.2.3. Measurement Process

The measurement process was confirmed based on the experimental scheme formulated in advance and the conditions on site. After completing the measurement for hairtail fish, the measurement for pork was started. We arrived at Suguo supermarket at 8:00 a.m. during the measurement period. First, 250 g of fish or pork prepared in advance was processed properly. Meanwhile, the electronic nose was switched on. Like the laboratory process, the measurements in a flow of pure air filtered by activated carbon were continued for 30 min to observe the sensor signal and to allow time for equilibration. By 8:30 a.m., the baseline had reached equilibration. Subsequently, the measurement started. The samples were analyzed at half day intervals until the samples spoiled, which took 3 days for hairtail fish and 5 days for pork. Every measurement was also repeated six times. Finally, according to the sensory evaluation and the previous results pattern in the laboratory, the change process in the quality of the test objects was analyzed.

## 3. Results and Discussion

### 3.1. Comparison Responses of Electronic Nose with TVBN and Total Number of Aerobic Bacteria

Moist fresh fish has almost no fishy odor. The fishy odor develops with time after harvest. Generally, the number of microorganisms on the skin and gill surfaces, known as specific spoilage organisms (SSO), increases gradually and spreads to various tissues when the fish die. Volatile compounds, such as TMA, DMA, and ammonia, are the by-products of the decomposition of protein, amino acids, and other nitrogen compounds by the microorganisms. These by-products are collectively known as TVBN. Water, protein, fat, and a few carbohydrates are the primary compounds of pork. All kinds of volatile gases are generated because of the work of the enzymes and bacteria, e.g., protein is decomposed into ammonia, H_2_S, and mercaptan; fat is decomposed into aldehyde and aldehyde acid; and carbohydrate is decomposed into alcohol, ketone, and carboxylic acid. Volatile compounds increase with pork spoilage. During each phase of storage, different volatile compounds are present. Hence, TVBN levels are potential indicators of fish and pork spoilage [28].

**Figure 3.**
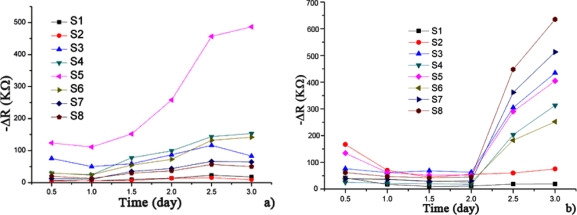
Time responses of the electronic nose for three days for hairtail fish (**a**) and pork (**b**).

The samples were introduced into the sample container successively. Figure 3 shows the opposite number of the D-value in the resistance (−ΔR), which represents the responses of the electronic nose to hairtail fish and pork samples stored at 15 °C for 3 days. The steady-state resistance minus the initial resistance of the sensors yields ΔR. The trends between the resistance (Figure 3) and concentration variations of the volatile compounds as hairtail fish and pork degrade are consistent. From these plots, which represent the resistance variation of the sensor array, a slight variation in resistance is observed in hairtail fish [Figure 3(a)] during the first two days of exposure. A sharp increase in resistance occurs in the time interval between two and three days. Finally, the responses show a tendency to stabilize. A difference exists in pork [Figure 3(b)] because of the difference in volatile compounds between hairtail fish and pork. A slight fluctuation occurred during the first two days. Between two and three days, the resistance of the sensors undergoes a sharp rise. Overall, the output signals of gas sensors for hairtail fish and pork gradually strengthened with extended storage. The electronic nose had a higher response to the dynamic headspace of the samples in an interval time of 1.5 days to three days.

**Figure 4.**
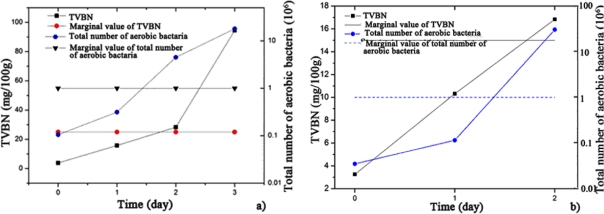
Changes in the TVBN and total number of aerobic bacteria during storage for hairtail fish (**a**) and pork (**b**).

Figure 4 describes the change in the TVBN and total number of aerobic bacteria with increasing storage days for the hairtail fish and pork samples. During approximately the first 1.5 days of exposure stored at 15 °C, the total number of aerobic bacteria for hairtail fish [Figure 4(a)] and pork [Figure 4(b)] were greater than standard values (10^6^ cfu/g). After approximately two days, TVBN exceeded standard values (for hairtail fish, 25 mg/100 g, and for pork, 15 mg/100 g). Comparing Figure 3 with Figure 4 shows that, for either hairtail fish or pork, the overall responses of the sensor array are generally consistent with the measurement of the TVBN and total number of aerobic bacteria.

**Figure 5.**
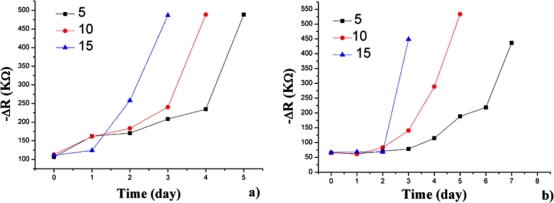
Time response of S5 at different temperatures for hairtail fish (**a**) and pork (**b**).

The present work was undertaken to compare the responses of the sensor array to the dynamic headspace of hairtail fish and pork samples stored at 15, 10, and 5 °C. The measurement of the hairtail fish and pork samples stored at 10 and 5 °C also draws the same conclusion. Figure 5 summarizes the information on the responses of the S5 (TGS 826, NH_3_, and amines) to hairtail fish and pork dynamic headspace during storage at 15, 10, and 5 °C. The overall trend is similar for different storage conditions. The spoilage rate is evidently most rapid at 15 °C, as noted by the fastest variation in the resistance of the sensor. Temperature at 10 °C decelerates the spoilage rate or the formation of volatile compounds. The variation in resistance of the sensor is lowest at 5 °C.

### 3.2. Modeling

**Figure 6.**
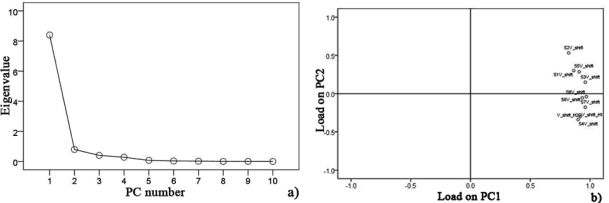
(**a**) Scree plot and (**b**) Load plot with PCA for hairtail fish samples at 15 °C.

**Figure 7.**
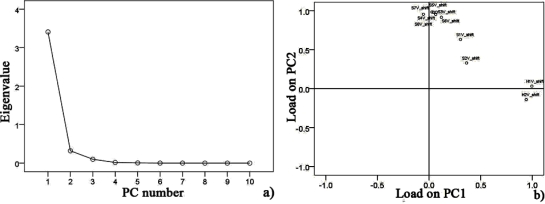
(**a**) Scree plot and (**b**) Load plot with PCA for pork samples at 15 °C.

Data collected at 15 °C after pre-processing, testing sphericity with KMO and Bartlett’s via SPSS Statistics 17.0 program showed that the KMO value is 0.752 over 0.7 for hairtail fish and 0.794 over 0.7 for pork. Hence, PCA could be performed as an unsupervised classification method to visualize the resemblance and difference among different measurements, including samples at different storage days and temperature in the datasets. The sensor signals were normalized when performing the PCA. Through this procedure, a set of N principal components were calculated. Figures 6 and 7 show the PCA results for hairtail fish and pork, respectively.

**Figure 8.**
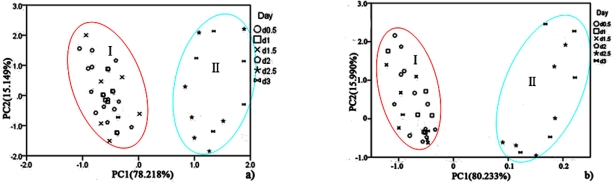
Score plots of PCA at 15 °C for hairtail fish (**a**) and pork (**b**) (without considering the temperature and humidity).

**Figure 9.**
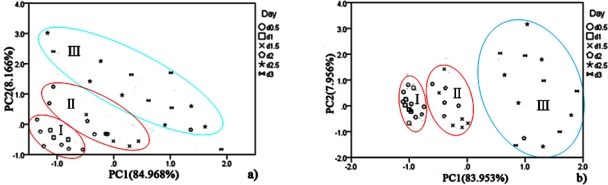
Score plots of PCA at 15 °C for hairtail fish (**a**) and pork (**b**) (direct compensation).

**Figure 10.**
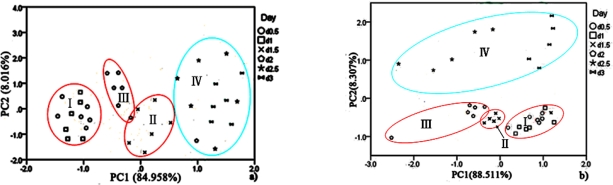
Score plots of PCA at 15 °C for hairtail fish (**a**) and pork (**b**) (PCA compensation).

The results of the PCA (Figures 6 and 7) show that the responses of the sensors are strongly correlated and two PCs can be extracted. In the process of application, gas sensors are sensitive to environmental temperature and humidity. During each phase of hairtail fish and pork storage, ambient temperature and humidity are not constant. Thus, the compensation of temperature and humidity is essential. Figures 8, 9, and 10 show the score plots of the data collected at 15 °C in the PC1 to PC2 planes for hairtail fish and pork. Direct compensation (Figure 9) and PCA compensation compensation (Figure 10) are adopted in the current work. Direct compensation is performed according to the temperature and humidity coefficient of the gas sensor. The formula is as follows:

(4) $$R=R_{0}-a\left(H-H_{0}\right)-b\left(T-T_{0}\right)$$

where R is the resistance after compensation, R_0_ is the measured resistance, H is the measured humidity, T is the measured temperature, H_0_ is the benchmark humidity (H_0_ = 0), T_0_ is the benchmark temperature (T_0_ = 25 °C), and a and b are the humidity and temperature coefficients of the gas sensor, respectively.

For hairtail fish, Figure 8(a) (without considering temperature and humidity) shows that the measurement cluster is classified into two different groups, I (fresh) and II (spoiled). The first group corresponds to samples having undergone up to two days of storage. The second group corresponds to samples that underwent from two days to three days of storage. Figure 9(a) (direct compensation) shows that the dataset is grouped into three, I (more fresh), II (fresh), and III (spoiled). The measurement corresponding to the first, second, and third storage days formed the first, second, and third groups, respectively. Figure 10(a) (PCA compensation) shows that the measurements cluster in four different groups, namely, I (most fresh), II (more fresh), III (fresh), and IV (spoiled). Every group underwent half-day of storage. Table 1 lists the aromas of each classification for hairtail fish and pork.

The same measurement dataset with different compensation yields different results. The same class samples may have a similar smell. Accordingly, these samples are located in the same region. By a comparison between Figures 8(a), 9(a), and 10(a), all fresh samples can be distinguished from the spoiled samples. However, Figure 10(a) could distinguish in more specific detail. For pork, the comparison between Figure 8(b) (without considering the temperature and humidity), Figure 9(b) (direct compensation), and Figure 10(b) (PCA compensation) could also lead to the same conclusion. The two principal components, PC1 and PC2, could be used to represent 93.367% of the data variance in Figure 8(a), 93.134% of the data variance in Figure 9(a), 92.974% of the data variance in Figure 10(a), as well as 96.223% of the data variance in Figure 8(b), 91.909% of the data variance in Figure 9(b), and 96.818% of the data variance in Figure 10(b).

Figures 8(a), 9(a), and 10(a) show the projections of the experimental results on a two-dimensional plane PC1–PC2. Thus, by contrast, the conclusion is easily drawn that the compensation for temperature and humidity is necessary and that PCA compensation is better, simple, and convenient. Factor-Figures of PCA could reflect the process of deterioration for hairtail fish and pork samples. In this case, the PCA method based on PCA compensation shows a good separation between fresh and spoiled hairtail fish and pork samples and could consequently be used to determine the shelf life of hairtail fish and pork rapidly.

**Figure 11.**
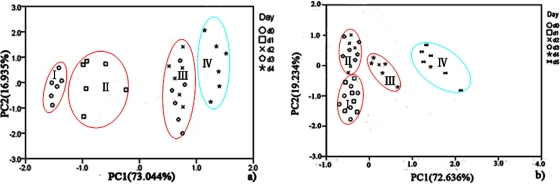
Score plots of PCA at 10 °C for hairtail fish (**a**) and pork (**b**) (PCA compensation).

**Figure 12.**
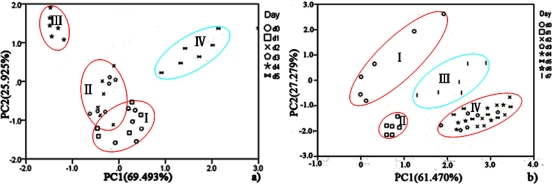
Score plots of PCA at 5 °C for hairtail fish (**a**) and pork (**b**) (PCA compensation).

PCA was employed to process all the data collected from different storage temperatures for hairtail fish and pork. Figures 11 and 12 show the PCA results based on the PCA compensation of hairtail fish and pork stored at 10 and 5 °C, respectively. The measurement cluster is grouped into four different parts, namely, I (most fresh), II (more fresh), III (fresh), and IV (spoiled). From these plots, the shelf life for hairtail fish stored at 15, 10, and 5 °C was 2, 3, and 4 days of storage, respectively, and the shelf life for pork stored at 15, 10, and 5 °C was 2, 4, and 6 days of storage, respectively. By comparing Figure 10 with Figures 11 and 12, it can be easily concluded that electronic noses can detect the spoilage rate of hairtail fish and pork samples increases with increasing storage temperature. The results of PCA based on PCA compensation are shown in Figures 10, 11, and 12, where PC1 and PC2 extracted through PCA varied at different temperatures. On one hand, the classification figures by PCA clearly distinguish between fresh and rotten samples, but their discrimination patterns are different. On the other hand, the gaps of each PC for different temperatures are smaller. For the hairtail fish samples, the PC at 15, 10, and 5 °C was 92.974% [Figure 10(a)], 89.979% [Figure 11(a)], and 95.418% [Figure 12(a)], respectively. For the pork samples, the PC at 15, 10, and 5 °C was 96.818% [Figure 10(b)], 91.870% [Figure 11(b)], and 88.749% [Figure 12(b)], respectively. The position of fresh and spoiled samples is close in the factor-figures of PCA under different temperatures. Although the spoiled tracks of hairtail fish and pork samples were different, the basic areas of fresh and spoiled samples in the factor-figure s of PCA under different temperatures were similar. Hence, to distinguish the fresh and not-fresh samples of hairtail fish or pork clearly, the discrimination patterns should be changed with the temperature.

### 3.3. Validation of the Model Based on Field Measurement

The hairtail fish was spoiled and could not be eaten by the second day, based on the sensory evaluation. According to consecutive tests and sensory evaluation, the pork samples were rotten by the fourth day. Hence, the shelf life of hairtail fish and pork in the supermarket was approximately two and four days of storage, respectively. For accurate results, the discrimination patterns should be ensured based on storage temperature. The average storage temperatures for hairtail fish and pork samples were close to 15 and 10 °C, respectively, according to the storage conditions in the supermarket. The shelf life of hairtail fish and pork samples stored at 15, 10, and 5 °C were determined in the laboratory. Hence, the discrimination pattern of 15 °C fit the hairtail fish sample, and the discrimination pattern at 10 °C was applied in analyzing the pork samples.

The discrimination patterns of 15 and 10 °C were used to process the data collected from hairtail fish and pork in the field measurement. First, we calculated the center of the ellipses in Figures 10(a) and 11(b). Subsequently, the data was applied to the corresponding model. If a point was near the center of an ellipse, we assumed that the point belonged to the ellipse. Finally, the results were compared with Figures 10(a) and 11(b) to obtain the analysis results. According to the abovementioned principle, the analysis results were listed in Tables 1 (for hairtail fish) and 2 (for pork). As shown in Figures 10(a) and 11(b), the tables list the number of points, which either fell into the corresponding ellipse (Correct) or did not fall into the ellipse (False). The points that represent the times of measurement are disparate to different storage days. Accurate rates also appear in the tables.

Tables 2 and 3 summarize the results of hairtail fish and pork classification when the discrimination patterns of 15 and 10 °C were implemented to estimate the accurate rate of monitoring and predicting the shelf life, respectively. The results show that the classification accuracy rate of hairtail fish is 87.5%, and the classification accuracy rate of pork can reach 91.7%. To a large extent, the results confirm that the electronic nose with PCA can generally classify the samples.

## 4. Conclusions

We report here on the development of a simple electronic nose based on an array of commercially available MOS gas sensors aimed at monitoring the freshness of hairtail fish and pork stored at 15, 10, and 5 °C in the laboratory. A dynamic headspace sampling method was also employed. TVBN and total number of aerobic bacteria analyses were applied as contrast methods. The results of the electronic nose were proven to be accurate, and the electronic nose had the advantages of rapid measurement and low cost. The electronic nose was then used to measure hairtail fish and pork freshness in a supermarket for the Blacksmith Camp community in Nanjing.

In the laboratory, the responses of the sensors correlated well with the classical TVBN and total number of aerobic bacteria measurements. For hairtail fish, correlation coefficients were 0.97 and 0.91, and for pork, correlation coefficients were 0.81 and 0.88, respectively. At different storage temperatures, the spoilage rates of the samples were different. Hence, we could build a recognition pattern according to the corresponding spoilage rate. The accuracy of the analysis of the process of hairtail fish and pork spoilage was improved. The sensor array coupled with PCA could be trained not only to distinguish between the fresh and rotten samples in real-time, but also to identify the storage days by testing the change in volatile components.

In an actual application, the shelf life of pork and hairtail fish was monitored and predicted by implementing the discrimination patterns of 15 and 10 °C by monitoring the freshness of the products that were part of the Suguo supermarket. However, this method is not very accurate. The classification accuracy rate of hairtail fish was 87.5%, and the classification accuracy rate of pork reached 91.7%. These results confirm that the electronic nose with PCA can generally distinguish the samples as I (most fresh), II (more fresh), III (fresh), or IV (spoiled).

Based on the results, it can be concluded that the electronic nose coupled with PCA built in our laboratory is a promising simple and rapid instrument for monitoring and predicting the shelf life of hairtail fish and pork samples. Although the accuracy was low, further studies will be conducted to optimize the sensor array and to determine the optimal capacity of the electronic nose in monitoring and predicting the shelf life of hairtail fish and pork.

## Figures and Tables

**Table 1.** Aromas of each classification for hairtail fish and pork.

**Category**	**I**	**II**	**III**	**IV**
**Hairtail fish**	No smell	No smell	Slight ammonia taste or acid	Strong ammonia taste or acid
**Pork**	No smell	No smell	Slight ammonia taste or acid	Ammonia taste or acid

I (most fresh), II (more fresh), III (fresh), and IV (spoiled).

**Table 2.** The analysis results of hairtail fish in field measurement (Sample Number = 24).

**Classification**	**Correct**	**False**	**Accurate rate (%)**
I	6	0	100
II	5	1	83.3
III	4	2	66.7
IV	6	0	100

***Total***	**21**	**3**	**87.5**

I (most fresh), II (more fresh), III (fresh), and IV (spoiled).

**Table 3.** The analysis results of pork in field measurement (Sample Number = 24).

**Classification**	**Correct**	**False**	**Accurate rate (%)**
I	6	0	100
II	5	1	83.3
III	5	1	83.3
IV	6	0	100

***Total***	**22**	**2**	**91.7**

I (most fresh), II (more fresh), III (fresh), and IV (spoiled).
